# Population Viability Analysis on Chinese Goral Indicates an Extinction Risk for a Local Population in Beijing, China

**DOI:** 10.3390/ani14071126

**Published:** 2024-04-07

**Authors:** Rihan Wu, Xin Zhang, Jianxi Zhao, Deying Yi, Fuli Gao, Weidong Bao

**Affiliations:** 1 National Engineering Research Center of Tree Breeding and Ecological Restoration, College of Biological Sciences and Technology, Beijing Forestry University, Beijing 100083, China; wurihan0219@163.com (R.W.); fuligao@bjfu.edu.cn (F.G.); 2 Beijing Miyun District Yunmengshan Forest Management Unit, Beijing 101506, China; zx739969302@163.com (X.Z.); a15120067390@163.com (J.Z.); 13671179510@163.com (D.Y.)

**Keywords:** Chinese goral, wildlife management, VORTEX model, supplementation, sensitivity analysis

## Abstract

**Simple Summary:**

The Chinese goral (*Naemorhedus griseus*) is listed as a national second-class key protected wild animal in China. It is the only high level protected wild ungulate in Beijing. However, it faces threats that include habitat fragmentation and the disappearance of local populations. Population viability analysis (PVA) is one of the main ways to project the future abundance of species and provide reliable information for developing effective protection strategies. Through a pilot study, we found that the studied population in Beijing Yunmengshan Nature Reserve will initially increase in the next 20 years and then decrease with a 32% probability of extinction risk. Supplementation would help this population survive. The results show that vital limiting factors include the initial population size, sex ratio at birth, and mortality of infants.

**Abstract:**

The Chinese goral (*Naemorhedus griseus*) is identified as a vulnerable species on the Red List of China’s Biodiversity and listed as a national second-class key protected wild animal in China. It is a representative flagship ungulate in Beijing. Its distribution range is fragmented and small populations are separated by dense infrastructures and tourism sites. Understanding its population status provides a foundation to plan effective conservation strategies. In this study, a population viability analysis was conducted with VORTEX (10.5.6.0) on a Chinese goral population in Beijing Yunmengshan Nature Reserve with the data collected by camera trapping and parameters referenced from other goral populations. The results show that this population will initially increase in the next 20 years and then decrease with a 32% probability of extinction risk. Supplementation with four adults, two females and two males, every 10 years would help minimize the extinction risk of this population. The results highlight the vital limiting factors for Chinese goral, including the initial population size, sex ratio at birth and mortality of infants (especially female infants). To improve the protection efficiency, detailed population parameters should be further acquired through continuous monitoring of this population. A thorough large-scale study should be carried out on other segregated goral populations in Beijing to facilitate the recovery of this endangered species.

## 1. Introduction

Population viability analysis (PVA) is widely used to predict future population changes and extinction risks through the use of existing population data of an endangered wildlife population [1]. This approach can also identify important factors impacting population survival probabilities by sensitivity analyses [2], and provide reliable information for developing effective protection and management strategies [3]. In the 1970s, Craighead et al. [4] and McCullough [5] used simulation models to evaluate the effect of management on the extinction probability of the brown bear (*Ursus arctos*) population in Yellowstone National Park, thereby accelerating the study of PVA models. Combined with the concept of the minimum viable population (MVP) [6], the use of PVA has developed rapidly and become one of the valuable tools in the study of wildlife conservation [7,8].

Software for various PVA models has been developed, including VORTEX, INMAT, GAPPS, RAMAS/Metapop, and RAMAS/Stage [1,9]. The choice of software is crucial depending on the circumstances, as mismatched selections can compromise the prediction accuracy. INMAT is typically employed for examining short-term inbreeding effects [10]. GAPPS, tailored for grizzly bears (*Ursus arctos horribilus*), addresses specific needs in large mammal studies [11]. RAMAS/Metapop is primarily used for metapopulation dynamics and is equipped with GIS functions [12]. RAMAS/Stage is suitable for simulating species with very high fecundities, particularly those with large population sizes like fish [13]. VORTEX, given its comprehensive capabilities, is tailored for vertebrates with extended life cycles and low fecundity [14]. VORTEX models can simultaneously balance population statistical randomness and environmental randomness including genetic randomness and disaster randomness. Additionally, this software considers the influence of age structure, density restriction, inbreeding depression, hunting, and supplementation, as a result of which it has been widely used in the population viability analysis of large-sized animals [1]. Since the PVA model was first applied on the Chinese river dolphin (*Lipotes vexillifer*) in the Yangtze River in the early 1990s [15], Chinese scholars have simulated the future population dynamics of many mammals, such as the water deer (*Hydropotes inermis*) [16], giant panda (*Ailuropoda melanoleuca*) [17], black and white snub-nosed monkey (*Rhiuopithecus hieti*) [18], moose (*Alces alces*) [19], red deer (*Cervus elaphus*) [20], sika deer (*Cervus nipponkopschi*) [21], golden snub-nosed monkey (*Rhinopithecus roxellana*) [22], South China tiger (*Panthera tigris amoyensis*) [23], and Père David’s deer (*Elaphurus davidianus*) [24]. These works have effectively facilitated wildlife conservation in China.

The Chinese goral (Naemorhedus griseus) is a national second-class key protected wild animal, listed in the CITES Appendix I (the Convention on International Trade in Endangered Species of Wild Fauna and Flora, www.cites.org.cn (accessed on 20 October 2023)), and was categorized as vulnerable in the Red List of China’s Biodiversity (www.mee.gov.cn (accessed on 20 October 2023)). Despite its widespread distribution across 21 provinces or autonomous regions in China [25], there has been limited research on the population ecology of this species. At present, there are reports on its preliminary genetic structure [26], activity rhythm [27], food habits [28], population viability analysis [29], and hormone level analysis [30]. Despite these efforts, a comprehensive understanding of gorals’ biology remains insufficient. The distribution range of the Chinese goral in Beijing is fragmented and small populations are separated by dense infrastructures and tourism sites. Population recovery is being severely hampered by urbanization. However, there are only a few studies on Chinese goral in Beijing, one of the main distribution areas of gorals. Therefore, additional research is urgently needed to assess the status and future population trends of the goral populations.

In recent years, Beijing municipality has increasingly strengthened the laws and regulations regarding ecological environment protection to secure its biodiversity [31]. However, basic ecological information on many key protected species is still lacking. The diurnal rhythm of the Chinese goral was identified as a crepuscular activity pattern by camera trapping in Beijing Wulingshan Nature Reserve [32]. The distribution range of the goral shows a recovering trend in Beijing Songshan National Nature Reserve [33]. Nonetheless, there was still a gap in research on the speices’ future population dynamics and extinction risk in Beijing.

The Yunmengshan Nature Reserve is one of the main population areas for the conservation of Chinese gorals in Beijing. Notably, in both 2020 and 2021, there was a positive recovery trend observed in the abundance of gorals within the reserve [34]. Due to the rapid development of ecological tourism around the reserve in recent years, human disturbance has aggravated habitat fragmentation. Thus, detailed research is essential to comprehensively grasp the population dynamics of this species and identify the significant limiting factors constraining future population growth. In this study, a population viability analysis of the Chinese goral population in Yunmengshan Nature Reserve was conducted using a VORTEX model incorporating data collected by camera trapping and parameters from other goral populations. Furthermore, the sensitivities of the limiting factors affecting population growth were tested in order to supply theoretical support for population management. Based on our previous field surveys and camera trap monitoring, we hypothesized that the goral population in this area is robust and not facing a risk of extinction in the predictable future.

## 2. Materials and Methods

### 2.1. Study Area

**Figure 1:**
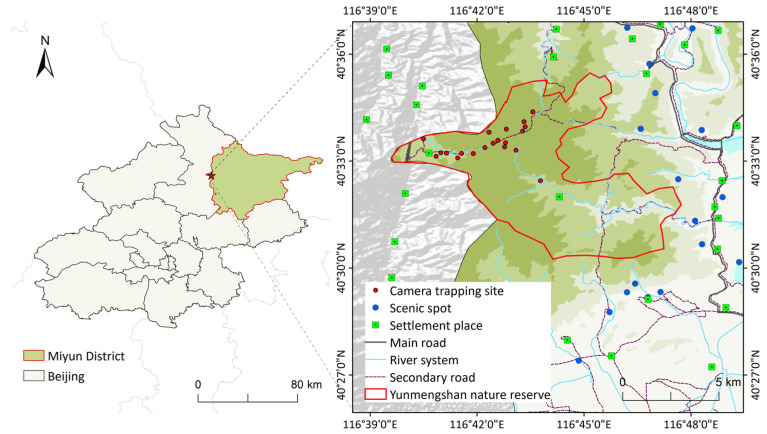
Study area and camera trapping sites in Beijing Yunmengshan Nature Reserve.

Beijing Yunmengshan Nature Reserve is located in the west of Miyun District, with the geographical location of 116°40′–116°47′ E and 40°30′–40°35′ N (Figure 1). The total area of the reserve is 4388 ha with a core area of 1019 ha. The Yunmengshan Nature Reserve is characterized by a mountain climate with an average summer temperature ranging from 20 °C to 24 °C and an average winter temperature ranging from −5 °C to −9 °C, creating a suitable environment for wildlife. Since July 2019, the entire reserve has been off-limits to tourists as part of biodiversity management efforts. Only hiking enthusiasts have occasionally entered the reserve along fixed climbing routes. Despite the closure, the reserve is bordered by numerous tourism sites, main roads, and villages, contributing to the isolation of the habitat for wild mammals within the reserve. Two biodiversity surveys conducted by field line transects and camera trapping in 2001 and 2017 recorded 36 species of mammals in this nature reserve. These two wildlife surveys allowed us to obtain the baseline information on the distribution ranges and population statuses of many species, including the Chinese goral. The commonly occurring mammals include rock squirrel (*Sciurotamias davidianus*), Asiatic striped squirrel (*Tamiops swinhoei*), Asian badger (*Meles leucurus*), hog badger (*Arctonyx collaris*), leopard cat (*Prionailurus bengalensis*), roe deer (*Capreolus pygargus*), and wild boar (*Sus scrofa*) [34].

### 2.2. Research Methods

VORTEX 10.5.6.0 (scti.tools/vortex (accessed on 6 October 2022)) was used to analyze the population viability of Chinese gorals in this study. We predicted the survival dynamics of the population over the next 100 years and repeated the simulation 1000 times to reduce the systematic error. We analyzed the effects of supplementation on population growth under different initial population sizes. Finally, sensitivity analysis was performed on the main parameters, including lethal equivalent (LE), sex ratio at birth in male (SR), mortality of infant females/males (MIF/MIM), mortality of adult female/males (MAF/MAM), effects of drought (ED), initial population size (IPS), and carrying capacity (K). Through the obtained sensitivity index, the most important factors that affect the population dynamic were selected to test population development trends.

### 2.3. Model Parameter Selection

#### 2.3.1. Inbreeding Decline

The VORTEX model used the lethal equivalent (LE) to estimate the effect of inbreeding depression. We used the average LE of 2.79 derived from 14 species of Artiodactylas instead of the default value to make our results more realistic [35].

#### 2.3.2. Reproductive System

The reproductive system of goral is polygynous [36,37], the breeding age was set to two years old for females and three for males [38], the maximum age of reproduction was set to 17, and the maximum lifespan was 18 [39]. Gorals usually enter estrus from October to November and give birth in April or May of the following year [40]. Usually each brood has one offspring, but in some cases there are two offspring [41]. The sex ratio at birth was set to 1:1 following the general rule of mammalian species [26] (Table 1).

**Figure 2:**
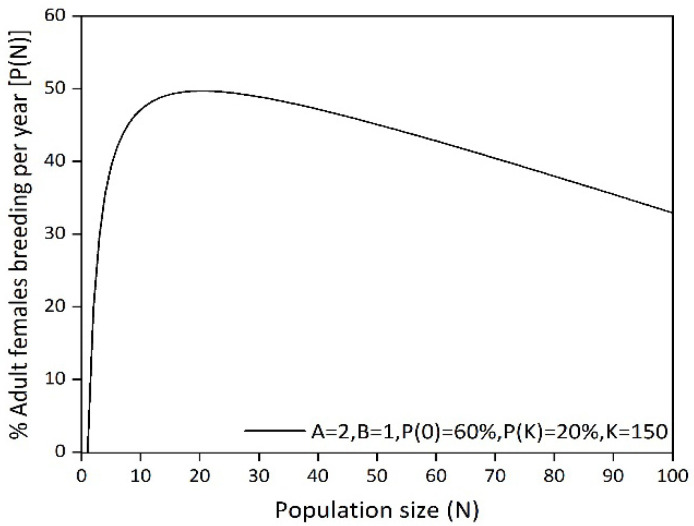
A density-dependent effect in female reproduction.

Due to the small area for the goral population in Yunmengshan Nature Reserve, we assume there is a density-dependent effect on female reproduction with the increase of the population size [29]. Using the function editor within VORTEX, we estimated the steepness parameter of the B value to be 1 and the A value of the Allee effect parameter, describing the decrease in female mating rate, to be 2 to better reflect the density-dependent effects of the gorals in this study (Figure 2) [42,43].

#### 2.3.3. Mortality Rate

The mortality rates of different ages determine the future trends of the population. We simulated the population dynamics of different ages under different mortality conditions. Due to the few records of the mortality cases of Chinese goral in the study area, we used the mortality parameters of the closely related species of long-tailed goral (*Naemorhedus caudatus*) in Korea [44] (Table 2).

#### 2.3.4. Catastrophe

The Chinese gorals use higher mountain areas as their preferred habitat. Floods have no negative influence on them, and mountain fires are strictly managed in the Beijing region, so drought would be the natural disaster most likely to threaten their life. Over the past two decades, Miyun District experienced a persistent nine-year drought where the annual precipitation from 2000 to 2009 was significantly below the multi-year average. Accordingly, we set the frequency of drought at 45% [45]. As this species is listed in the second class of state key protected wildlife, rescue management would be carried out if there was a severe drought emergency. In this study, we estimated that drought would reduce the reproduction rate by 1% and survival rate by 2% under the situation of rescue management.

#### 2.3.5. Initial Population Structure

In this study, the population abundance was estimated by a random encounter model (REM) based on modelling random encounters between moving animals and static camera traps [46]. In our previous studies conducted from April to November in 2020, we utilized camera trapping and GPS collaring for data collection to estimate the initial goral population size [34]. Every two hours, we acquired the GPS signals of the collared gorals to obtain precise data on their average speed [47]. With random placement, 21 cameras were positioned within the core area, covering an area of 752.29 ha. The effective detection distance of the cameras was 7.5 m. In order to reduce autocorrelation, we placed cameras at distances exceeding 200 m from each other to prevent duplicate counts (Figure 1). All computation processes were implemented using the “cameratrapR” package [48]. This approach relied on capturing frequent detections, thereby increasing our ability to maximize the estimation of the highest population size. Gorals were infrequently active beyond the core area; therefore, it is inappropriate to assume that densities in these regions were comparable to those in the core area. We chose a maximum estimate of goral abundance in the non-core area of about half of that observed in the core area. Using the initial population size of 78 in the reserve, we analyzed the impact of different initial population sizes (halving and doubling, i.e., IPS = 39 and 156) on future population dynamics. Because the gorals have similar appearances between males and females and between adults and sub-adults, we could not determine the age distribution of the studied population. The age-sex structure was automatically generated using the stable age distribution function in the VORTEX model.

#### 2.3.6. Carrying Capacity

We estimated the carrying capacity under ideal conditions by using the home range of gorals in Yunmengshan Nature Reserve [29]. As the reserve is in a closed state for conservation, we used the whole area of 4388 ha for our estimation. We calculated the average home range size of Chinese gorals tracked in the study area (95% MCP = 0.33 km^2^, *n* = 5) using the Global Messenger 3.03 platform (data.hqxs-tracker.com (accessed on 30 October 2023)). Considering the habitat quality, spatial behavior, and overlap index of home ranges [49], the carrying capacity of the nature reserve was set to K = 200.

#### 2.3.7. Supplementation

Given the low dispersal rate of surrounding individuals into the study area, we initially set the supplementation intervals to once every 10 years, with the numbers of females and males ranging from 1 to 10 and in an increment of one animal every year.

### 2.4. Minimum Viable Population

Minimum viable population (MVP) is a quantitative measure that defines the smallest population size that has a 90% probability of surviving for 100 years [6]. The MVP of the goral population was estimated by an asymptotic approximation method [50].

### 2.5. Sensitivity Analysis

The sensitivity index can be used to determine the degree of impact of the parameters on the dynamics of the population [51]. We used the sensitivity testing function within VORTEX to systematically assess the impact of each factor used in the model. Initially, we assessed the most influential parameters using a limited sample size, refining parameter ranges accordingly. Subsequently, employing a single-factor sampling method, we simulated population dynamics in diverse environments. The existing parameter values were used as the baseline, and then each parameter was adjusted up or down by approximately 50%, with 1000 repetitions for each setting [52]. Through analysis of the relationship between the changes in parameters and the sensitivity or extinction probability, we identified the important factors affecting the future abundance of the goral population.

## 3. Results

### 3.1. Population Survival over the Next 100 Years

Based on the VORTEX model, when the initial population size (IPS) was 78, the deterministic growth rate (r) of the goral population was 0.0489, the finite growth rate (λ) was 1.0501, the net reproductive rate (R_0_) was 1.3926, and average female and male generation time was 6.77 and 5.52 years, respectively. The mean population generation time was 6.15 years, the probability of extinction (PE) was 32%, and the mean time to extinction (TE) was 69.42 years.

**Figure 3:**
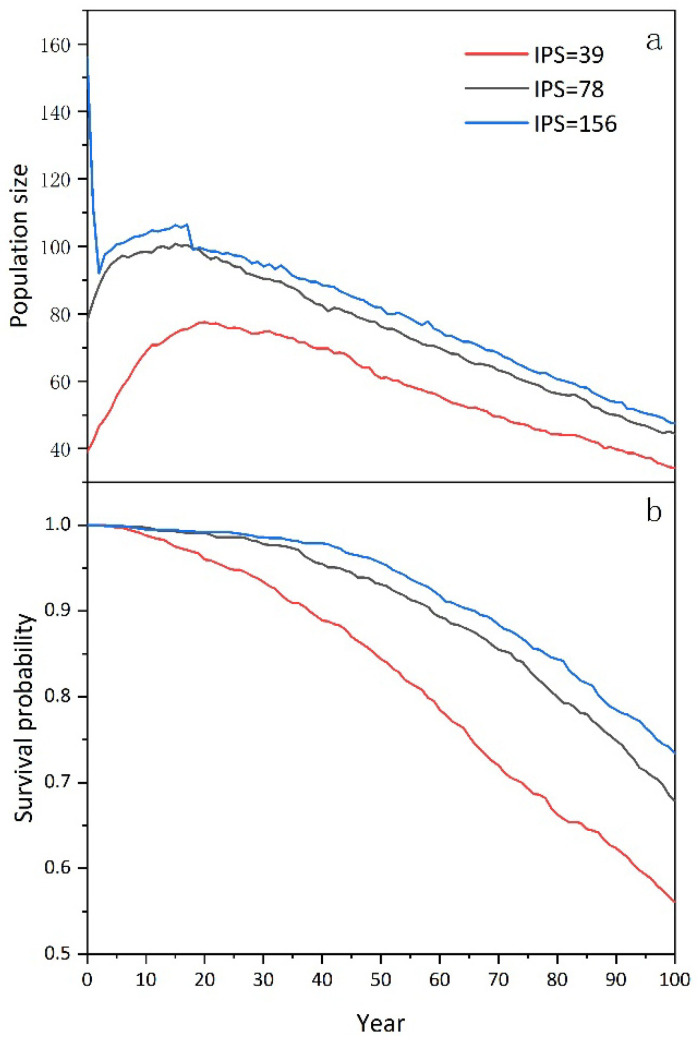
The general variation in population size (**a**) and survival probability (**b**) of Chinese goral with different initial population sizes (IPSs) in the next 100 years in Beijing Yunmengshan Nature Reserve.

Under different IPSs, the future population size (N) and survival probability (PS) changed differently. When the IPS was 39 and 78, the population size increased during the first 20 years and then decreased, whereas at the 100th year, the final population size decreased to 34 and 44, respectively. When the IPS was set to be 156, the population decreased in the first 5 years, then increased untill the 20th year, and then decreased again. By the 100th year, the final population size was 47, which was much lower than the initial 156 (Figure 3a). The PS of the studied goral population showed an overall downward trend no matter how big the IPS was (Figure 3b): the smaller the IPS, the greater the decline and the higher the PE for the studied population.

**Figure 4:**
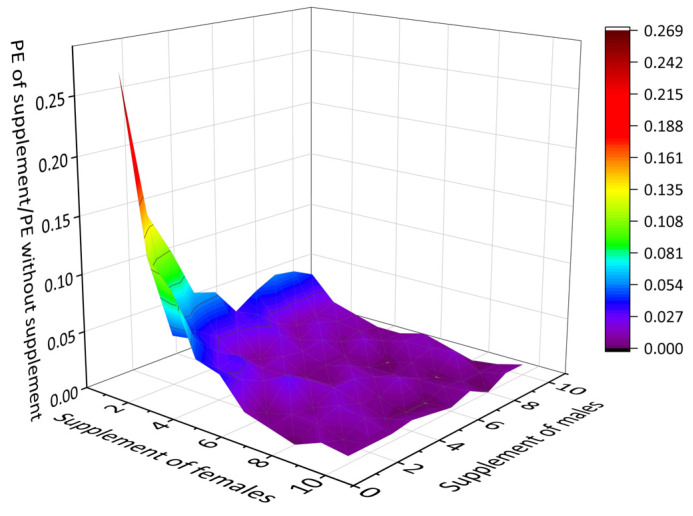
Effects of supplementing females and males at 10-year intervals on the probability of extinction. The baseline for this supplementation aligns with the parameters of the existing population at IPS of 78.

The estimated MVP that could maintain a 90% survival rate in 100 years for this goral population was 110. Model predictions indicated that supplementing the population every 10 years would significantly decrease the PE, if not entirely eliminating it. Regression tests demonstrated a substantial influence of the supplemental females on the PE, highlighting that a higher proportion of females was more beneficial for the PS. If four adults, comprising two females and two males, were supplemented every 10 years, the PE would decrease to zero. Consequently, the stochastic population growth rate and final population size would surpass those of the population without supplementation (Figure 4).

### 3.2. Sensitivity Analysis

**Figure 5:**
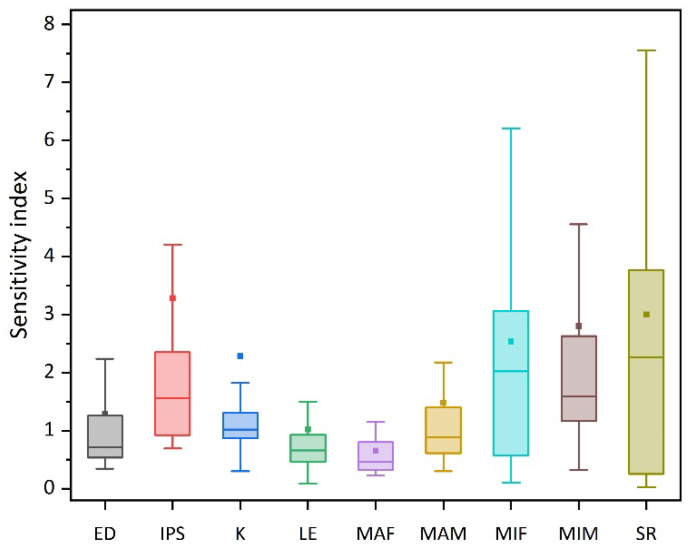
Sensitivity analysis on different factors affecting the dynamics of the goral population in Beijing Yunmengshan Nature Reserve (ED: effects of drought, IPS: initial population size, K: carrying capacity, LE: lethal equivalents, MAF/MAM: mortality of adult female/males, MIF/MIM: mortality of infant females/males, SR: sex ratio at birth in male).

Among the parameters affecting the goral population dynamics, the sensitivity index of the IPS was 3.2848 ± 6.2858, which indicated that the IPS was the most critical factor as its sensitivity index was the highest. The sensitivity indices of SR, MIM, and MIF were 2.9982 ± 3.8517, 2.8039 ± 3.5484, and 2.5388 ± 2.9790, respectively, also indicating the importance of these factors in affecting the population growth (Figure 5).

#### 3.2.1. Sex Ratio at Birth in Male (SR)

**Figure 6:**
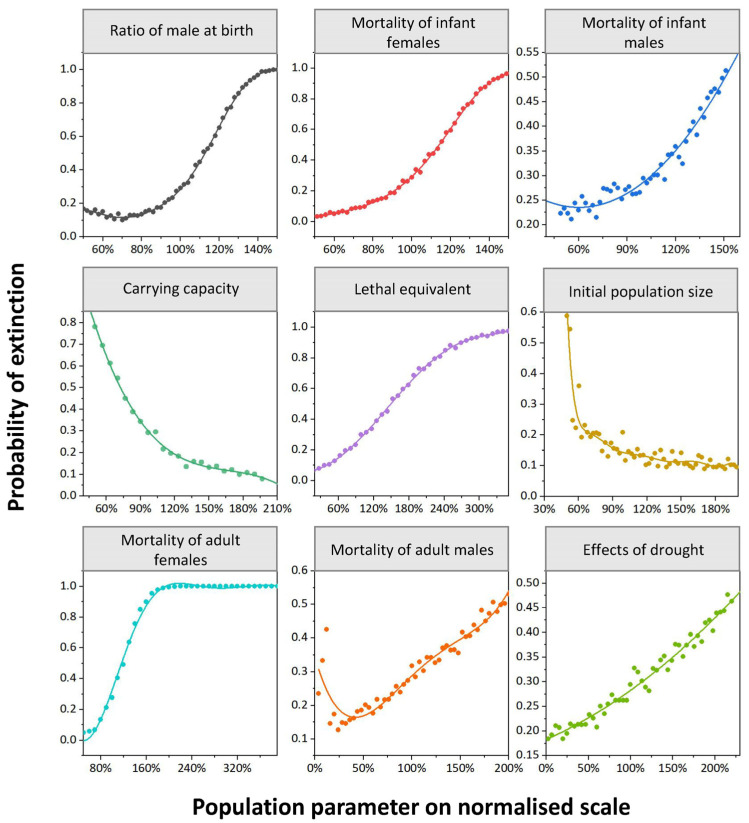
Parameters that influence the extinction probability of the goral population in Beijing Yunmengshan Nature Reserve.

In this goral population, as the SR increased, the population showed a high PE. When the SR was input at 75%, which was 140% of the current SR, the PE increased to 100% (Figure 6). This result indicates that the value of the SR has a greater negative impact on the population growth and its sensitivity index is high.

#### 3.2.2. Mortality of Infants (MIF/MIM)

After reducing or increasing the MIF and MIM by 50%, it was found that the infant mortality had a greater impact on the population growth. When the MIF and MIM were lower, the PE decreased significantly and the stochastic population growth rate increased (Figure 6).

#### 3.2.3. Carrying Capability (K)

Halving or doubling K, the growth rate of the population size changed significantly. When the K was halved, the PE increased to 90%, and when the K was doubled, the PE declined to 10%, suggesting that the sensitivity of the carrying capacity was high (Figure 5 and Figure 6).

#### 3.2.4. Lethal Equivalent (LE)

In this study, the LE was used to simulate the inbreeding decline of the goral population. As the LE increased, the PE also increased. When the LE was 10, the PE increased to 100% (Figure 6). The result shows that inbreeding accelerates the extinction rate of the goral population.

#### 3.2.5. Initial Population Size (IPS)

The IPS had the highest impact on this goral population (Figure 5). If the IPS decreased from 200% to 60%, the PE would increase correspondingly. Moreover, when the IPS dropped below 60% of the current IPS, that is, when the IPS was lower than 47 animals, the PE was close to 100% (Figure 6).

#### 3.2.6. Mortality of Adults (MAF/MAM)

After reducing or increasing the MAF and MAM by 50%, it was found that the MAF has a greater impact than the MAM on the population growth. With an increase in MAF to 180%, the PE reached 100%. However, with a similar increase in MAM to 180%, the PE only reached 50% (Figure 6).

#### 3.2.7. Effects of Drought (ED)

After running different catastrophe parameters of drought, the results showed that the PE would increase if the frequency of drought increased (Figure 6).

## 4. Discussion

In recent years, the habitat suitability for many wild animals in China has gradually decreased due to infrastructure and urban expansion, resulting in an increased risk of population extinction in China [53]. As a biodiversity-rich capital, Beijing has established many nature reserves to safeguard the endangered wild animals in their natural habitats, yet it still faces increasing pressure to enhance the conservation of wildlife and its habitats [54]. Fortunately, through the implementation of effective protection measures, wild animal populations are increasing, and some key protected animals that have not appeared for a long time have been recorded. For example, the reappearance of Chinese goral at the China National Botanical Garden in 2022 [55] indicates that the conservation efforts have produced positive effects.

The Chinese gorals have been observed in many provinces in China, but there are few conservation studies on this species, with only one report on population viability analysis in Inner Mongolia [29]. Due to the constrained habitat conditions for gorals, the fragmentation of suitable habitats, and the considerable distances between isolated reserves, the goral populations within each reserve in Beijing are largely separated, resulting in limited dispersal events [26]. The extinction of isolated populations could reduce their genetic diversity, potentially aggravating the risk of extinction for gorals. Hence, comprehending the dynamics of these isolated populations is crucial for formulating effective conservation strategies. Furthermore, with the low population sizes, the potential extinction risk for these populations is high. The threatening factors identified in our study for the Beijing Yunmengshan Nature Reserve population can provide insights to prevent the extinction of other populations in China. The results of our study suggested a 32% probability of extinction for the goral population in the Beijing Yunmengshan Nature Reserve over the next century. Therefore, effective conservation measures are imperative to mitigate the extinction risk and ensure population viability.

In this study, we have simulated the population trends and assessed the major impact factors for a goral population in Beijing (Figure 3 and Figure 5). The results indicate that a smaller initial population size (IPS) corresponds to a lower future population size and reduced probability of survival. Based on the initial population size of 78 determined from camera trapping data, the population appears to be recovering currently, which is partially consistent with our assumption at the beginning of the study; however, there is still a long-term extinction risk. Research findings indicate that the REM model demonstrates a high degree of credibility in estimating ungulate population abundance [47,56], particularly when assessing rare species exhibiting cryptic behaviors [57]. The estimated initial population size in this study might be inflated owing to the dense goral concentration in the core area where the cameras were distributed. Our assumption that the population density in the non-core area was half that of the core area might also lead to an overestimation for the initial population size. Nevertheless, even in the presence of a potential overestimation of the initial population size, it is crucial to acknowledge that this goral population remains under significant threat of extinction. In this context, our results provided valuable insights into the protection of the goral population. Recognizing the importance of data accumulation for metrics like population size [58], inbreeding effects [37], and reproduction rates [59], continuous monitoring and data collection in subsequent studies are crucial to enhance the reliability of simulation outcomes.

We found that the estimated initial population size of 78 is still lower than the estimated MVP and the survival probability is only 68%. Therefore, it is necessary to develop appropriate conservation measures to assure an effective recovery for this goral population. Additionally, our analysis of the MVP suggests that long-term survival of the goral population could be achieved by expanding the population size. The changes in population size with different IPSs show that augmenting the IPS could increase the population growth, but when the IPS increases to a certain point, the predicted population exhibits a downward trend. The reason for this phenomenon may be a limitation in habitat quality, such as the increased competition from density-dependent effects that result in a shortage of subsistence resources (Figure 2). Consequently, in order for the goral population to be stable and persist long-term, it is imperative to maintain the habitat quality of the reserve and to enlarge the suitable living space if possible.

To facilitate the recovery of the goral population and mitigate the risk of extinction, we have added additional supplementation measures. In exploring the impact of the sex ratio and the number of supplements on the PE, we recommend a supplementation interval of 10 years—a cost-effective approach designed to provide practical insights for planning conservation programs. Ideally, if supplementing four adults consisting of two females and two males every 10 years, the extinction rate would reduce to zero (Figure 4). Our results indicated that supplementation of exogenous individuals could almost eliminate the extinction risk. Studies on Canadian bighorn sheep (*Ovis canadensis*) have also demonstrated that genetic diversity could be restored and population adaptability could be increased by supplementation [60]. The notable report on Przewalski’s horse (*Equus przewalskii*) emphasized the significance of introducing non-related individuals in rescuing small groups [61].

The stable age distribution in the VORTEX 10.5.6.0 was used to automatically generate the ratios of age and sex in the population. The generated male-female ratio was about 1:1.6 which is similar to the sex ratio of 1:1.53 in the Chinese goral population in Beijing Songshan Nature Reserve [26], demonstrating that the automatically generated sex ratio has reference value and can be used to simulate the actual population. There being more female Chinese gorals is related to their polygynous reproductive system and is also a manifestation of population growth. We suggest that it is necessary to conduct molecular monitoring on the sex ratio of this goral population for effective conservation.

Based on our sensitivity analysis, there are many factors affecting the survival of the goral population, with the IPS, SR, and MIM/MIF being the most critical factors (Figure 5). Population augmentation can be achieved through the introduction of exogenous individuals, while safeguarding pregnant females and progenies can contributes to the recovery of the population. Our results show that the habitat’s carrying capacity has a great influence on population growth. We suggest that it is necessary to protect the living space, both the habitat’s quantity and quality, to promote population recovery. In addition, the results also suggest that females contribute more to population changes than males in this goral population (Figure 6), which is also supported by the PVA conducted on the goral population in Inner Mongolia [29]. Accordingly, conservation efforts should be implemented to maintain the habitat quality such as supplying food and salt licks during the winter and breeding season when food is scarce to ensure the survival of pregnant females and their progenies.

## 5. Conclusions

Population viability simulation can provide many meaningful insights into factors limiting population growth that may be difficult to identify in field studies. This can strengthen our ability to plan practical conservation strategies for endangered species. In our study, VORTEX 10.5.6.0 was used to analyze the population viability and the sensitivity of parameters in an effort to reveal the determinant and random factors affecting a Chinese goral population in Beijing Yunmengshan Nature Reserve. Based on the existing initial population size of 78, the extinction risk of this goral population is 32% and the average extinction time is 69.42 years even though the population will increase in the first 20 years. This result is partially supported by our field survey that this population has been increasing recently, but its future fate is uncertain. Therefore, practical conservation measures are needed to avoid population decline. It is necessary to carry out supplementation measures with four adults (including two females and two males) every 10 years. Meanwhile, suitable habitats should be expanded by enlarging areas around the nature reserve to improve the carrying capacity for this population. This measure would also promote the spreading out of the younger individuals to reduce the negative effects of inbreeding. During the harsh winter and the spring breeding seasons, appropriate feeding measures should be considered to ensure the survival of juveniles and female adults, thus promoting effective reproduction and the long-term recovery of this Chinese goral population.

## Figures and Tables

**Table 1:** Reproductive parameters used in PVA on Chinese goral in Beijing Yunmengshan Nature Reserve.

Parameter	Value
Reproductive system	Polygamous
Age of first offspring for females	2
Age of first offspring for males	3
Maximum age of reproduction	17
Maximum lifespan	18
Maximum number of broods	2
Sex ratio at birth	1:1

**Table 2:** Mortality schedule used in PVA on Chinese goral in Beijing Yunmengshan Nature Reserve.

Age Class	Mortality (SD) ^1^ (%)	
	Female	Male
0~1	45 (17)	45 (17)
1~2	20 (10)	35 (15)
2~3	10 (10)	25 (15)
>3	10 (10)	25 (15)

^1^ SD: Standard deviation.

## Data Availability

The data that support the findings of this study are available from the corresponding author upon reasonable request.
